# Effects of dynamic bedroom lighting on measures of sleep and circadian rest-activity rhythm in inpatients with major depressive disorder

**DOI:** 10.1038/s41598-022-10161-8

**Published:** 2022-04-12

**Authors:** Markus Canazei, Johannes Weninger, Wilfried Pohl, Josef Marksteiner, Elisabeth M. Weiss

**Affiliations:** 1 Department of Psychology, University of Innsbruck, Innrain 52f, 6020 Innsbruck, Austria; 2 Research Department, Bartenbach GmbH, Rinnerstrasse 14, 6071 Aldrans, Austria; 3 Abteilung Psychiatrie und Psychotherapie A, Regional Psychiatric Hospital, 6060 Hall in Tirol, Austria

**Keywords:** Psychology, Human behaviour

## Abstract

Bright light therapy is an effective treatment option for seasonal and non-seasonal affective disorders. However up to now, no study has investigated effects of dynamic bedroom lighting in hospitalized patients with major depression. A bedroom lighting system, which automatically delivered artificial dawn and dusk and blue-depleted nighttime lighting (DD-N lighting) was installed in a psychiatric ward. Patients with moderate to severe depression were randomly assigned to stay in bedrooms with the new lighting or standard lighting system. Patients wore wrist actimeters during the first two treatment weeks. Additionally, hospitalization duration and daily psychotropic medication were retrieved from patients’ medical charts. Data from thirty patients, recorded over a period of two weeks, were analyzed. Patients under DD-N lighting generally woke up earlier (+ 20 min), slept longer (week 1: + 11 min; week 2: + 27 min) and showed higher sleep efficiency (+ 2.4%) and shorter periods of nighttime awakenings (− 15 min). In the second treatment week, patients started sleep and the most active 10-h period earlier (− 33 min and − 64 min, respectively). This pilot study gives first evidence that depressed patients’ sleep and circadian rest/activity system may benefit from bedroom lighting when starting inpatient treatment.

## Introduction

Altered alertness and disturbed circadian rhythms as well as sleep problems are often reported in persons with affective disorders^1^ and may play a crucial role in the pathogenesis of this disease^2^.

Light acutely alters vigilance in humans^3^. Timed light exposure additionally has the potential to resynchronize the circadian clock^4^ and to improve sleep quality^3^. Thus, timed light exposure is a promising non-pharmacological intervention option in the treatment of affective disorders.

Today, light therapy is a first-line treatment for seasonal affective disorders^5^ (SAD), and may be used in treating patients with moderate to severe depression^6,7^. Moreover, combined medication and light therapy showed greater efficacy than pharmacotherapy alone in depressed patients^6^. Despite these promising results, light therapy is still rarely applied in clinical settings today.

Light therapy comprises a daily morning, polychromatic white light exposure of 5000 lx-hours, is fast-acting in its antidepressant effect^1,8^, and has a minor side-effect profile (e.g., headache, eyestrain, nausea, and agitation) occurring in up to 45% of patients within the first treatment days^9^.

In 1989, Terman et al.^10^ conducted a pioneering study and reported beneficial effects of artificial dawn and dusk in three SAD patients. Later, two landmark studies provided evidence that artificial dawn is as effective as light therapy in the treatment of SAD^11,12^.

Dawn simulators are installed at the bed head-side and deliver timed, gradually increasing light intensities in the early morning. They differ in several technical aspects to light therapy devices: light exposure occurs during the sleep–wake transition period over a time span of 30 min to 3 h and light intensities are gradually rising to outdoor sunrise illuminances between 250 to 1000 lx and peaking at the time of planned awakening (applied light dose in dawn simulation is thus substantially lower than in light therapy). Dawn simulators are further perceived as a more natural light intervention, deliver light without side-effects, and thus may be easier and more convenient to use than light therapy devices^13^.

Research in healthy people has broadened our understanding of artificial dawn effects: it improves sleep inertia^14–16^ and cognitive performance after awaking^15,17^. In addition, artificial dawn induces some physiological effects, e.g., a phase advance of the melatonin rhythm^18^, an accelerated decline in skin temperature^16^ and a reduced heart rate gradient during sleep–wake transition^19^.

Besides dawn, dusk is a second important natural cue for photic entrainment feeding into the evening oscillator of the biological clock^20^. Although the pioneering study of Terman et al.^10^ used artificial dawn and dusk, effects of artificial dusk were scarcely investigated. To the best of our knowledge only Danilenko and Hommes^21^ have compared effects of a rectangular lights on/off pulse with a dusk simulation in the evening in healthy subjects, showing reduced body movements in the first 40 min of sleep but no phase shifting of the circadian clock under artificial dusk.

Moreover, up to now, only two field studies have investigated effects of a combined artificial dawn and dusk intervention^22,23^. In these studies, persons with dementia living in nursing homes were exposed to artificial dawn and dusk that was fitted to individual sleep times. In a first study^22^, persons with dementia had an earlier sleep onset, shorter sleep latency, longer sleep duration and decreased nighttime activity after three intervention weeks. In a second study^23^, patients with dementia reported better mood upon awakening under artificial dawn and dusk. However, in this study no effects were observed on actigraphically measured circadian activity rhythm and sleep parameters.

In addition to light therapy and artificial dawn and dusk, nocturnal lighting may affect humans. Research has shown that blue-depleted nighttime lighting may reduce nighttime arousal prior to bedtime^24^ and minimize disruptions of circadian and sleep parameters in healthy people^25–27^.

It is worth noting that blue-depleted nighttime lighting can also be generated by wearing blue-light blocking glasses. In a recently published systematic review^28^ it was shown that this intervention decreased manic symptoms in bipolar patients but delivered inconclusive results in improving mood in patients with major depression. However, clear evidence was found in this review of blue-light blocking glasses for a reduction in sleep latency in patients with sleep disorders.

So far, research has shown beneficial effects of artificial dawn in SAD patients and of artificial dawn and dusk in patients with dementia. No study has yet investigated effects of dynamic bedroom lighting, consisting of a dawn-dusk simulation and blue-depleted nighttime lighting, in hospitalized patients with affective disorders.

The present prospective pilot study closes this gap. Outcome measures were actigraphically recorded sleep and circadian rest-activity rhythm parameters and clinical health data (i.e., medication and length of hospitalization). Based on current knowledge, we hypothesized an improved sleep quality (i.e., shorter sleep onset latency and higher sleep efficiency) and more stable circadian rest-activity cycles in hospitalized patients residing under this dynamic bedroom lighting.

## Methods

### Study population

Study participants were patients of a psychiatric hospital with an ICD-10 diagnosis of affective disorder (F32, F33, and F34; ICD-10^29,30^). Moreover, patients had to suffer from moderate to severe depression at admission, quantified with a score of at least 35 at the Beck Depression Inventory (BDI-V)^31^. Patients showing acute suicidal ideation at admission were excluded from study participation.

Clinical effects of light therapy can be seen in SAD patients after the first treatment week^13,32^. Even for patients with non-seasonal depression, light therapy induces a strong reduction of symptoms within two weeks^6^. Based on these results and the fact that depressed patients stayed at the psychiatric ward for an average of 17.5 days ± 7.9, we decided to include only patients with an inpatient treatment duration of at least for 14 days.

In total, 187 patients were asked for study participation at admission by a psychiatrist over the course of one year. Many of these patients did not meet inclusion criteria or declined to participate (n = 129). Consequently, 58 patients were randomly assigned to the two interventions (DD-N lighting: n = 29; standard lighting: n = 29). Data from 15 patients were lost to follow-up (due to unwillingness to wear the actimeter; all of these patients wore the actimeter only during the first two treatment days) and 13 patients stayed less than 14 days in the hospital (DD-N lighting: n = 6; standard lighting: n = 7). Thus, data from 30 patients were finally subjected to data analyses.

**Figure 1:**
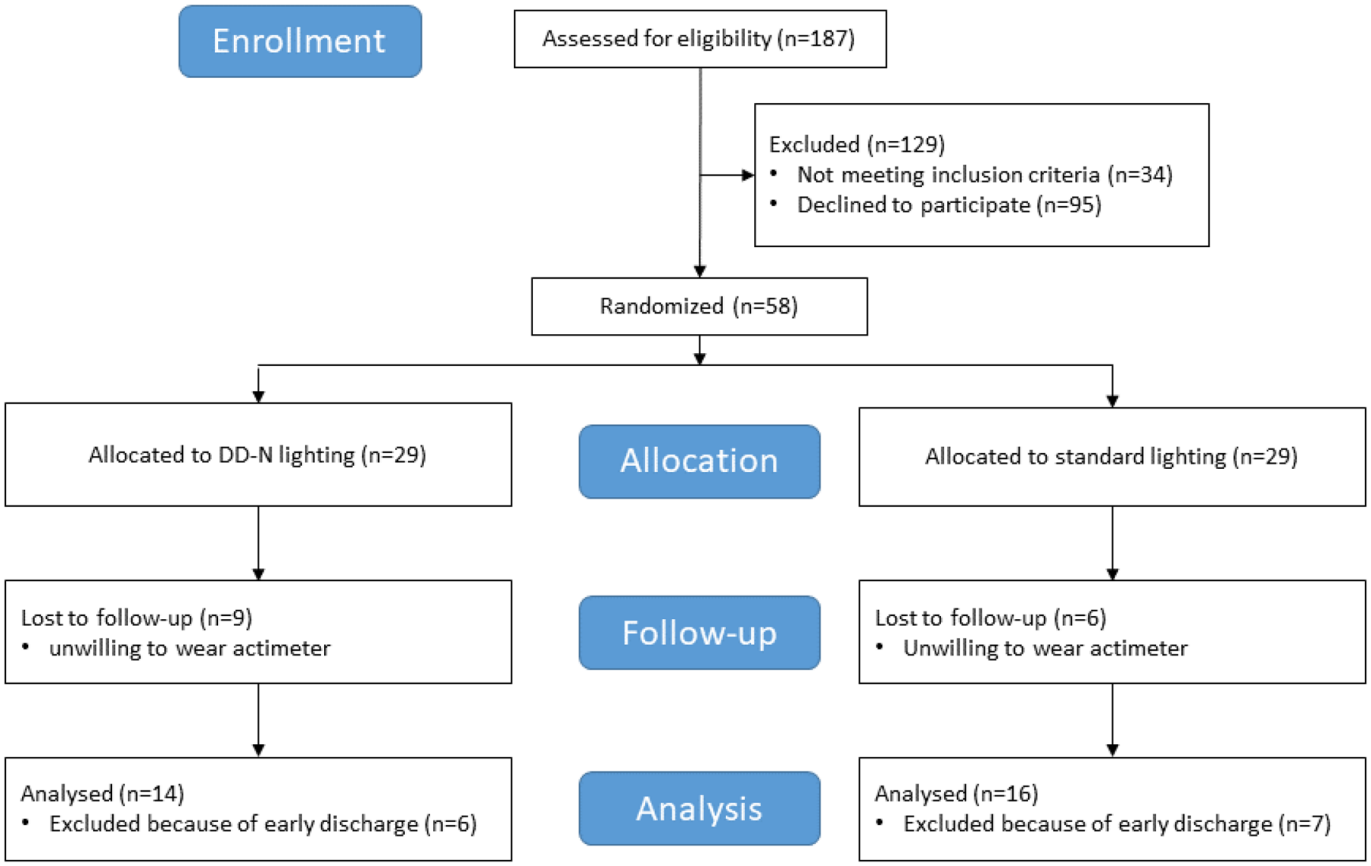
Study flow diagram.

A detailed study flow diagram of the present study is given in Fig. 1.

A total of 16 and 14 patients stayed in bedrooms with standard lighting and DD-N lighting, respectively. Gender distribution, mean age, distribution of ICD-10 diagnoses, and mean depression score (BDI-V) did not differ between the two groups (see Table 1).

**Table 1:** Description of study sample.

	Patients in bedrooms with standard lighting (n = 16)	Patients in bedrooms with DD-N lighting (n = 14)	Statistics
**Gender**			
Female	5	8	*p* = 0.153
Male	11	6	
Age (years)	44.6 ± 10.4	48.4 ± 14.1	*p* = 0.363
**ICD-10 diagnoses**			
F32 (depressive episode)	2	1	
F33 (recurrent depressive episode)	7	2	
F34 (persistent mood disorder)	7	11	*p* = 0.125
BDI-V	62.2 ± 17.0	57.2 ± 14.0	*p* = 0.444

Values are either shown as mean ± standard deviation or count data.

*BDI-V* Beck Depression Inventory, *ICD-10* International Classification of Diseases^29^.

Written informed consent was given by all subjects prior to study inclusion. The study protocol strictly followed the guidelines outlined in the 1964 Declaration of Helsinki and was approved by the ethical committee of the Medical University of Innsbruck, Austria.

### Descriptions of study bedrooms and lighting systems

#### Dimensions of the bedrooms and daylight penetration in the bedrooms

All four study bedrooms (two bedrooms with DD-N lighting and standard lighting, respectively) had the same room area (length: 4.60 m; width: 2.9 m), were located on the second floor and oriented to the east. Each bedroom had one window with an area of approximately 1.60 square meters. Tall, densely grown trees reduced daylight levels in the bedrooms and direct sunlight penetration in the morning. The daylight factor (i.e., the amount of daylight, measured horizontally at a height of 80 cm with a luxmeter at 1-m distance from the window in the bedroom on overcast days) in all four bedrooms was low (1.2% ± 0.8).

#### Bedrooms with artificial dawn-dusk and blue-depleted nighttime lighting (DD-N lighting)

In two double bedrooms in a psychiatric ward, seven tunable-white LED luminaires (WS14-01 FFF LED wallwasher, Projektleuchten GmbH, Dortmund, Germany) which could be tuned in color temperature and light intensity were installed at the upper wall opposite to the patients’ beds (see Supplementary Materials Fig. S1). Each luminaire comprised 24 LEDs (12 Luxeon Z-ES 2200 Kelvin and 12 Luxeon Z-ES 4000 Kelvin). In addition, bedside reading lights (research prototypes, Projektleuchten GmbH, Dortmund Germany), containing 10 LEDs (Luxeon Z-ES 2200 Kelvin), were installed in the bedrooms as well (see Supplementary Materials Fig. S2). To guarantee a consistent lighting design for the whole private area of the patient, the adjacent bathroom lights were further equipped with tunable-white LED luminaires (research prototypes, Projektleuchten GmbH, Dortmund Germany), providing the same light colors at the same daytime period as the bedroom lights. Ambient bedroom and bathroom luminaires were DALI-dimmable and controlled by a central lighting control system (Tridonic GmbH Co KG, Dornbirn, Austria). The reading light was manually adjustable in its intensity by a dimmer installed nearby the bed.

The lighting control strategy was aligned to the daily life of the patients in the psychiatric ward. Specifically, patients leave their beds at 07:00 h (or were woken by the staff at the latest at 07:00 h), so daytime activities (e.g., morning toilet and dressing) typically start at 07:00 h, followed by breakfast at 7:30 h (served in common rooms outside the bedrooms). Typically, patients reenter their bedrooms during short therapy breaks in the morning and afternoon and after lunch, but stay in public clinic areas during the day otherwise. The bedroom is again the main place of residence until after dinner at around 18:00 h. Commonly, patients get into their beds between 20:00 h and 21:00 h and read, watch TV or relax. Usually, patients start sleeping after the last caring procedures is finished at 22:00 h.

In line with these social routines and to generate a smooth light-induced sleep–wake transition period by ambient bedroom lighting, in the present study artificial dawn began 35 min before daily activities started (at 06:25 h) and was implemented with the ambient lighting system. During artificial dawn, light intensities gradually increased from 0 to 209 lx (horizontally at bed’s height) accompanied by risen color temperatures from 2192 to 3588 Kelvin generated by the two types of LEDs in the luminaires. At 07:00 h, the light intensity and light color was at its maximum. To stop artificial dawn, patients had to leave their beds, as the light switch was installed at the entrance of the bedroom.

During the day (from 07:00 h to 20:00 h), ambient bedroom lighting must be switched manually and provided 209 lx at bed’s height with a color temperature of 3588 Kelvin. After 20:00 h, ambient room lights were again dimmed automatically (artificial dusk) in its illuminance and color temperature over a period of 2 h. At 22:00 h, room lights provided 100 lx at bed’s height with a color temperature of 2192 Kelvin. In case, patients preferred to use reading lights for zonal illumination of the bed-side area, they were able to stop the artificial dusk at any time. Reading lights provided 20 lx at bed’s height with 2192 Kelvin and could be manually dimmed.

If necessary, room lights could be switched on during the night at any time and provided 100 lx at bedside level with 2192 Kelvin.

**Figure 2:**
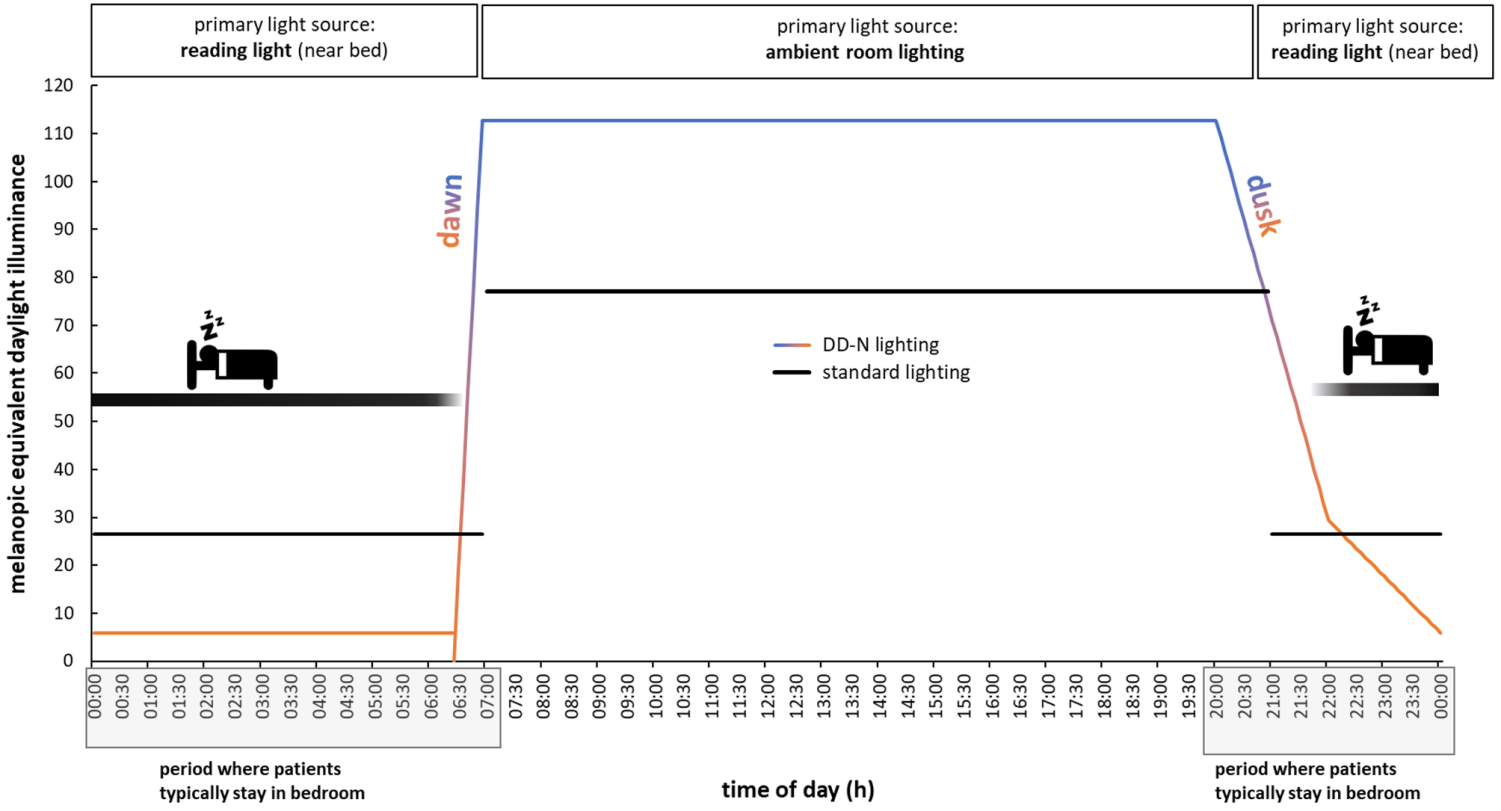
Light control strategies. Nighttime (00:00 h–06:25 h) and daytime (07:00–20:00 h) melanopic daylight-equivalent illuminances were reduced by 78% and increased by 46% under DD-N lighting compared to standard bedroom lighting, respectively. The lighting control system actually changed the light intensity and light color during dawn and dusk linearly over time (dawn: 6:25–07:00 h; dusk: 20:00–00:00 h).

The lighting control strategy for DD-N lighting is graphically summarized and compared to standard bedroom lighting in Fig. 2. The length of artificial dawn and dusk was not based on natural twilight periods. In particular, artificial dawn was significantly shorter (35 min) than natural dawn (i.e., the period from the beginning of the astronomic twilight to the end of the civil twilight) and aimed at preventing the patients from waking up prematurely. On the other hand, artificial dusk was significantly longer (4 h) than natural dusk to let patients decide sleep onset for themselves.

A comprehensive summary of photometrical measures generated by the two light interventions is given in Table 2.

**Table 2:** Photometric characterization of the lighting interventions.

Lighting system	Standard bedroom lighting		DD-N lighting			
	Ambient light	Reading light	Ambient light			Reading light
Timing	07:00 h–21:00 h *	21:00 h–07:00 h *	06:25 h–07:00 h	07:00 h–20:00 h	20:00 h–22:00 h	22:00 h–06:25 h
**Color properties**						
correlated color temperature	3513 Kelvin	3513 Kelvin	2192 Kelvin	3588 Kelvin	2192 Kelvin	2192 Kelvin
color rendition (CRI)	86	86	82	82	83	82
CIE coordinates (x,y)	(0.41, 0.40)	(0.41, 0.40)	(0.51, 0.42)	(0.41, 0.40)	(0.51, 0.42)	(0.51, 0.42)
**Illuminances (mean ± SD; lx)**						
horizontal, at bed level	140 ± 14	52 ± 4	20 ± 2	209 ± 21	100 ± 10	20 ± 2
vertical, at eye level of patient—20° tilted	131 ± 15	48 ± 5	19 ± 2	196 ± 23	94 ± 11	19 ± 2
vertical, at eye level of patient—70° tilted	131 ± 17	57 ± 7	20 ± 2	208 ± 21	98 ± 11	20 ± 2
**Luminances (range; cd/m**^**2**^**)**						
at opposite wall	29–41	5–23	3–10	27–98	15–49	3–10
at ceiling	21–345	5–98	2–75	25–780	13–394	2–75
**α-opic equivalent daylight (D65) illuminance (lx)**						
S-cone-opic	63.20	21.76	2.83	88.29	14.13	2.83
M-cone-opic	129.69	44.66	14.32	178.58	71.59	14.32
L-cone-opic	151.71	52.25	20.70	208.52	103.49	20.70
rhodopic	92.04	31.70	7.73	129.94	38.66	7.73
melanopic	77.10	26.55	5.88	112.83	29.41	5.88

Spectral irradiances and illuminances were measured with a spectroradiometer (specbos 1211-2, JETI Technische Instrumente GmbH, Jena, Germany) and color properties were calculated with the software JETI LiVAl; luminances were measured with a calibrated CANON EOS 650D camera with fisheye optic; the alpha-opic irradiances were derived from the spectral irradiance measurements excel-toolbox S026-2018 provided by the CIE.

*Typical switching times of ambient lights and reading lights were determined from staff’s observations.

#### Standard bedroom lighting

**Figure 3:**
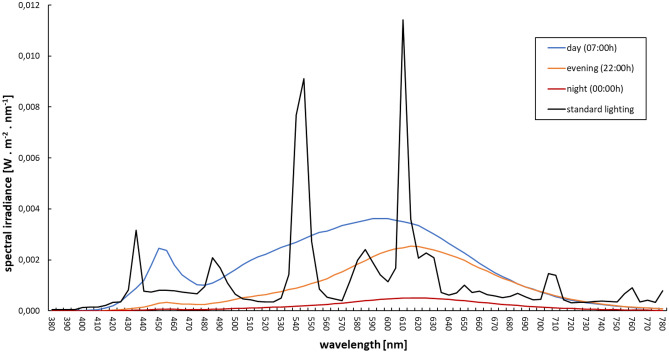
Light spectra in bedrooms with standard lighting and DD-N lighting. Note: black = standard lighting; blue, orange, and red = DD-N lighting.

Lighting design in the standard double bedrooms followed European lighting standards for hospitals (EN DIN 12464-1) and was implemented with two standard luminaires (‘Pureline Basic’, Zumtobel AG, Dornbirn, Austria), which were installed at the wall above the patient`s head. Each luminaire housed two fluorescent lamps (Osram T16 39W/840 G5 HO). Reading lights comprised the same fluorescent lamp technology and offered 52 lx horizontally (measured at the height of the bed). Spectral data of all relevant lighting conditions are given in Fig. 3. Following recommendation from Spitschan^33^, spectral raw data of the two light interventions are summarized in Supplementary Materials Table S3.

### Outcome measures

#### Wrist actimetry

The rest-activity cycle is a core behavioral measure of the circadian system and often disturbed in depressed patients^34^. An actimeter is a body-worn device, which comprises an accelerometer and allows the measurement of physical activity over periods of days or weeks. By means of these activity data sleep quality parameters^35^ and circadian activity rhythm parameters^36^ can be calculated. Due to the fact that sleep parameters derived from actimetry are similar to polysomnography (validation shows high sensitivity but moderate to low specificity^37^), this method has often been employed in sleep and light impact research^38^.

In the present study, we used a wrist-worn, light-weight (19 g), water-resistant device (ActiGraph wGT3x-BT; Pensacola, FL, USA). The device was worn on the non-dominant hand and recorded three-dimensional activity data with a sample rate of 30 Hz. To minimize non-wearing times, the actimeter was attached with a non-removable textile bracelet. We used the software ActiLife (Version 6.8.1; https://actigraphcorp.com/actilife/) to retrieve and process actimetry data (i.e., we aggregated data over time periods of 1 min) and to run the Cole-Kripke sleep scoring algorithm^39^. For the sleep analysis, usually a daily bedtime protocol is needed. In the present study, bedtimes were estimated from activity data: the individual starting time in bed was defined as the beginning of the latest 10-min period between 20:00 h and 23:00 h with lower physical activity compared to the mean daytime activity level of the subject; the individual end time in bed was defined as the end of the earliest 10-min period between 06:00 h and 8:00 h with lower activity compared to the mean daytime activity level of the ongoing day. The following sleep parameters were derived for each night: total sleep time (TST; hours), sleep onset latency (SOL; minutes), sleep efficiency (SE; percent), number of wake episodes (WASO; count data) and sleep onset and offset times (clock times). For statistical analysis, we calculated mean values of these sleep parameters separately for the first and second treatment week. To derive circadian activity rhythm parameters, we applied a non-parametric analysis routine^40^ to weekly activity data and calculated the following parameters also separately for each of the two treatment weeks: intra-daily variability (IV), inter-daily stability (IS), relative amplitude (RA), most active 10-h period (M10), clock time when M10 starts (M10 onset), least active 5-h period (L5), and clock time when L5 starts (L5 onset).

#### Hospital stay and psychotropic medication

Length of hospitalization and psychiatric medications (i.e., antidepressant, antipsychotic, and sedative medication) were retrieved from the patients’ medical chart. To compare treatments with different antidepressants, we calculated equivalent doses referring to citalopram^41^. A similar procedure was applied to antipsychotic medication^42^ and sedative medication^43^ with the reference agents Chlorpromazine and Diazepam, respectively. Due to the fact that pro re nata (PRN) medication was used occasionally (on 28 out of 420 treatment days) in few study participants, we did not extract PRN medication data from patients’ health records.

#### Non-pharmacological treatments

To control for non-pharmacological treatment regimens of patients in the two intervention groups, daily non-pharmacological treatments (i.e., ergotherapy, physiotherapy, and psychotherapy) were retrieved from the patients’ medical chart as well.

#### Light exposure during hospital stay

The lighting control system was not able to store usage times of the reading lights and manual switching maneuvers of the general room lights. However, the actimeter ActiGraph wGT3x-BT included a light sensor and thus made it possible to continuously record illuminances of up to 5000 lx at the patients’ wrist levels. Unfortunately, the light sensor has not yet been validated and recorded light data provided only an approximate measure of the patient's light exposure due to an inadvertently covering of the sensor with long-sleeved clothing or the blanket and the possibility to manually switch off room lights. To give an estimation for time periods with a covered light sensor, we report the percentage of time during waking periods with wrist illuminances smaller or equal to 1 lx. Notwithstanding, recorded light data at wrist level was used to estimate differences in daytime-specific light exposure levels between the two intervention groups.

#### Study protocol and data collection procedure

Patients were asked to participate in the study by a psychiatrist upon admission. After giving written informed consent, study participants wore an actimeter continuously for the first 14 days in hospital. Patients staying in bedrooms with DD-N lighting were further informed about the lighting control strategy and instructed how they can manually interrupt the automatic dimming procedure at any time using the light switches.

After handing in the actimeters after treatment day 14, the patients did not have to leave their bedroom but stayed there until the end of their hospital stay.

Physical activity and light exposure data as well as data from the patients’ medical charts for the first 14 treatment days were subjected to data analyses.

Overall, study participants were recruited over a period of one year.

### Statistical analysis

Descriptive statistics are given as mean and standard deviation (mean ± SD) and figures show averages and 95% confidence intervals of means. To compare demographic variables, length of hospitalization, and non-pharmacological treatments between the two intervention groups either a t-test, chi-squared test or exact Fisher test were run. Two-way mixed analysis of variances (ANOVAs) with the between-subjects factor light intervention and the within-subjects factor treatment week were used for the analysis of actimetric parameters, antidepressant and antipsychotic medication, and light exposure. Due to the fact that data of equivalent doses of sedative medication differed on the first treatment day, a mixed ANOVA, controlled for the equivalent dose of sedative medication on the first treatment day, was run. Effect sizes (partial eta-squared) are given for significant effects. All analyses were run with a significance level of 0.05 (two-sided).

By means of the software package G*Power^44^ (Version 3.1.9.4; https://www.psychologie.hhu.de/arbeitsgruppen/allgemeine-psychologie-und-arbeitspsychologie/gpower), a sensitivity analysis for a repeated-measures, within-between interaction model was run. For this model and under usual statistical assumptions (power = 80%, alpha = 0.05, correlation among repeated measurements = 0.5, and non-sphericity correction epsilon = 1), a sample size between 16 to 34 subjects allows the detection of moderate intervention effects^45^. This effect size was also found in light therapy studies with people with depressive disorders^6,7^.

## Results

### Sleep-related effects

#### Total sleep time (TST)

We found a large interaction effect between the two factors light intervention and treatment week on TST, *F*(1, 28) = 6.650, *p* = 0.015, partial η^2^ = 0.192. In the first week, TST was significantly higher under DD-N lighting (493.96 min ± 13.11) compared to standard lighting (483.37 min ± 13.08), with a mean difference of 11 min, F(1,28) = 4.880, p = 0.036, partial η^2^ = 0.148. In the second week, TST was again significantly higher under DD-N lighting (504.15 min ± 26.28) compared to standard lighting (477.58 min ± 18.62), with a mean difference of 27 min, F(1,28) = 10.408, p = 0.003, partial η^2^ = 0.271. In addition, TST significantly increased over time under DD-N lighting only, *F*(1, 13) = 5.476, *p* = 0.036, partial η^2^ = 0.296.

#### Sleep onset latency (SOL)

We could not observe any intervention effect on SOL, all *p* > 0.10.

#### Sleep efficiency (SE)

A mixed ANOVA revealed a significant main effect of intervention on SE, F(1,28) = 7.626, *p* = 0.010, partial η^2^ = 0.214, with higher SE under DD-N lighting (92.59% ± 2.82) compared to standard lighting (90.18% ± 2.91). The interaction effect and main effect of measurement time was not significant, both *p* > 0.10.

#### Wake after sleep onset (WASO)

WASO was significantly lower under DD-N lighting (38.70 min ± 12.71) compared to standard lighting (54.14 min ± 11.97) across both treatment weeks, with a mean difference of 15 min, F(1,28) = 23.557, *p* < 0.001, partial η^2^ = 0.968. Statistical analysis did not yield a significant interaction nor main effect of time on WASO, both *p* > 0.10.

#### Sleep onset time

There was a statistically significant interaction between the factors light intervention and time of measurement on sleep onset time, *F*(1, 28) = 4.312, *p* = 0.047, partial η^2^ = 0.133. In the first week, sleep onset times did not differ between DD-N lighting (21:48 h ± 41 min) and standard lighting (21:59 h ± 23 min), p > 0.10. However, in the second week sleep onset time was significantly earlier under DD-N lighting (21:28 h ± 28 min) than under standard lighting (22:01 h ± 33 min), with a mean difference of 33 min, F(1,28) = 8.642, *p* = 0.007, partial η^2^ = 0.236. In addition, patients under DD-N lighting started sleep earlier in week 2 compared to week 1, *F*(1, 13) = 5.428, *p* = 0.037, partial η^2^ = 0.295.

#### Sleep offset time

Independent of the treatment week, patients woke up earlier in the morning under DD-N lighting (06:35 h ± 20 min) compared to standard lighting (06:55 h ± 24 min), with a mean difference of 20 min, F(1,28) = 8.141; *p* = 0.008, partial η^2^ = 0.225. We further observed no significant interaction and main effect of measurement time on sleep offset time, both *p* > 0.10.

Interaction plots for all sleep parameters are given in the Supplementary Materials, Fig. S4. In addition, sleep data for the first three treatment days were compared between the two intervention groups (see Supplementary Materials, Table S5), indicating that actimetric sleep data was comparable between the two groups within the first inpatient treatment days.

### Circadian rest-activity rhythm effects

#### Intra-daily variability (IV) and Inter-daily stability (IS)

We could not observe any intervention effect on IV and IS, all *p* > 0.10.

#### Relative amplitude (RA)

RA significantly differed between the two treatment weeks, F(1,28) = 7.229, *p* = 0.012, partial η^2^ = 0.205, with lower RA in the week 1 (0.937 ± 0.030) compared to week 2 (0.946 ± 0.026). The interaction effect and main effect of intervention on RA was not significant, both *p* > 0.10.

#### Most active 10-h (M10)

Similar to RA, we could reveal a significant time of measurement effect on M10, F(1,28) = 27.127, *p* < 0.001, partial η^2^ = 0.492, with lower M10 in week 1 (2108 ± 572) compared to week 2 (2455 ± 788). Again, the interaction effect and main effect of intervention on M10 was not significant, both *p* > 0.10.

#### Onset time of M10

A two-factor mixed ANOVA showed a significant interaction between the factors light intervention and measurement time on the onset time of M10, F(1,28) = 9.305, *p* = 0.005, partial η^2^ = 0.249. Onset times of M10 did not differ in treatment week 1, *p* > 0.10, but in treatment week 2, F(1,28) = 11.793, *p* = 0.002, partial η^2^ = 0.296, with earlier onset time under DD-N lighting (06:43 h ± 32 min) compared to standard lighting (07:47 h ± 63 min), a mean difference of 64 min. In addition, under both bedroom lighting systems, onset time of M10 changed significantly over time: for standard lighting the onset time was delayed in week 2 (07:47 h ± 63 min) compared to week 1 (07:13 h ± 57 min), F(1,15) = 4.608, *p* = 0.049, partial η^2^ = 0.235. In contrast, under DD-N lighting M10 onset time significantly advanced from treatment week 1 (07:12 h ± 61 min) to treatment week 2 (06:43 h ± 32 min), F(1,13) = 5.297, *p* = 0.039, partial η^2^ = 0.289.

#### Least active 5-h (L5)

We could not reveal any intervention effect on L5, all *p* > 0.10.

#### Onset time of L5

Interaction between the factors light intervention and measurement time on onset time of L5 reached significance, F(1,28) = 4.250, *p* = 0.049; partial η^2^ = 0.132. Post-hoc analyses revealed no differences in the onset times of L5 between the two interventions in treatment week 1, *p* > 0.10, but a significant difference in treatment week 2, F(1,28) = 4.321; *p* = 0.047, partial η^2^ = 0.134, with an earlier onset time under DD-N lighting (23:10 h ± 55 min) compared to standard lighting (23:49 h ± 46 min), with a mean difference of 39 min. Under both interventions, onset times of L5 did not differ between the two treatment weeks: both *p* > 0.10.

In the Supplementary Materials S6a and S6b, interaction plots for effects on circadian rest-activity rhythm parameters are shown.

### Length of hospitalization

The length of hospitalization was not different between the two interventions: standard lighting (21.4 days ± 3.4) and DD-N lighting (20.3 days ± 5.8), t(28) = 1.074, *p* = 0.851.

### Pharmacotherapy

#### Antidepressant medication

An independent-samples t-test was run to determine if there were differences in antidepressant medication on the first treatment day between the two intervention groups. The equivalent daily dose of Citalopram did not differ at the beginning of inpatient treatment for patients under DD-N lighting (20.82 mg ± 14.13) and standard lighting (20.74 mg ± 14.12), *p* > 0.10.

Moreover, patients were prescribed a similar mean daily equivalent dose of Citalopram across the 14 treatment days with an average of 27.67 mg ± 13.68 under DD-N lighting and 27.59 mg ± 14.18 under DD-N lighting, *p* > 0.10. The daily equivalent dose of Citalopram, however, increased from 35.46 mg ± 29.67 in the first treatment week to 40.07 mg ± 35.40 in the second treatment week, F(1,28) = 4.453; *p* = 0.044; partial η^2^ = 0.137. We could not detect a significant interaction between the factors of light intervention and measurement time, *p* > 0.10.

#### Antipsychotic medication

Ten patients in bedrooms with DD-N lighting and 9 patients in bedrooms with standard lighting were treated with antipsychotic medication during the first two treatment weeks. Those patients were prescribed a similar mean daily equivalent dose of Chlorpromazine of 105.95 mg ± 64.36 under DD-N lighting and 109.96 mg ± 89.59 under standard lighting, *p* > 0.10. Again, neither the interaction between the two factors nor the main effect of measurement time of daily equivalent doses of Chlorpromazine were significant, both *p* > 0.10.

#### Sedative medication

Fourteen patients in each intervention group were treated with Benzodiazepines across the 14 treatment days. About half of this type of medication (43–51%) was given daily in the evening and night (17:00–22:00 h). An independent-samples t-test showed that the equivalent daily dose of Diazepam were significantly different at the first treatment day in patients under DD-N lighting (7.44 mg ± 8.44) compared to standard lighting (20.03 mg ± 17.89), *t*(26) = 9.264, *p* = 0.025. Thus, a mixed ANOVA, controlled for the equivalent dose of Diazepam at treatment day 1, was run. We could not detect a difference between the two light intervention groups in the daily equivalent dose of Diazepam (DD-N-lighting: 7.56 mg ± 6.21; standard lighting: 8.71 mg ± 6.11; *p* > 0.10). In addition, the interaction between the two factors light intervention and measurement time were not significant, *p* > 0.10. However, we observed a main effect of time with a significant higher daily dose of Diazepam in the first treatment week (10.91 mg ± 4.16) compared to the second treatment week (5.37 mg ± 4.77), F(1,26) = 28.650; *p* < 0.001; partial η^2^ = 0.534.

### Confounder analysis

#### Non-pharmacological treatments

Following a multimodal treatment approach, all subjects took part in ergo-therapies, physiotherapies, and psychotherapies on work days from 09:00 h to 11:00 h and 14:00 h to 16:00 h. The daily mean number of therapies did not differ between the two groups over the 14 treatment days; DD-N lighting: 2.5 therapies ± 0.5 and standard lighting: 2.5 therapies ± 0.4, t(28) = 0.185, *p* = 0.767. Additionally, both groups participated equally in the therapies: DD-N lighting: 20% ergo-therapy, 69% physiotherapy, and 11% psychotherapy; standard lighting: 18% ergo-therapy, 71% physiotherapy, and 11% psychotherapy, *p* = 0.882.

#### Light exposure across different daytime periods

##### Light exposure during the artificial dawn period (06:25 h to 7:00 h)

The proportion of time after awakening spent under illuminances smaller or equal to 1 lx were 18.2% under DD-N lighting and 16.7% under standard lighting. As expected, mean illuminance level at wrist level was higher under DD-N lighting during automatic dawn (43.86 lx ± 6.00) compared to standard lighting (20.47 lx ± 5.6), F(1,28) = 8.099, *p* = 0.008; partial η^2^ = 0.224. We could not find a significant interaction nor main effect of time of measurement on illuminance in the early morning, both *p* > 0.10.

##### Daytime light exposure (07:01 h to 19:59 h)

During the day, the portion of time periods with recorded illuminances smaller or equal to 1 lx were 1.2% for patients staying in bedrooms with DD-N lighting and 1.1% for patients staying in bedrooms with standard lighting. Mean daytime wrist illuminance levels did not differ (*p* > 0.10) and were 200.41 lx ± 72.80 for subjects under DD-N lighting and 212.37 lx ± 70.07 under standard lighting. In addition, daily exposure duration above 1000 lx were also equivalent between the two groups; DD-N lighting: 50 min ± 14; standard lighting: 49 min ± 13, *p* > 0.10.

##### Light exposure during the artificial dusk period (20:00 h to 22:00 h)

The proportion of time before falling asleep with very low illuminances (i.e., smaller or equal to 1 lx) were 12.2% under DD-N lighting and 14.8% under standard lighting. Wrist illuminance levels were significantly lower under DD-N lighting (24.32 lx ± 28.31) compared to standard lighting (48.93 lx ± 33.60) during the dusk period, F(1,28) = 6.826, *p* = 0.014; partial η^2^ = 0.196. The interaction effect and main effect of treatment week did not reach significance, both *p* > 0.10.

##### Nighttime light exposure (22:01 h to 06:24 h)

Nighttime light levels were significantly lower under DD-N lighting (0.61 lx ± 1.73) compared to standard lighting (1.63 lx ± 1.95) in the first treatment week, *p* = 0.003, and also in the second treatment week; DD-N lighting: 0.81 lx ± 2.34; standard lighting: 2.16 lx ± 2.41, *p* = 0.019. The interaction and time effects on nighttime illuminance measured at wrist level did not reach significance, both *p* > 0.10.

## Discussion

The present study aimed at investigating dynamic ambient bedroom lighting effects in moderately to severely depressed patients at the beginning of inpatient treatment using wrist actigraphy and data from patients’ medical charts. The bedroom lighting system significantly increased morning (from 6:25 h to 7:00 h) and decreased evening and nighttime light exposure (from 20:00 h to 6:25 h) compared to standard bedroom lighting. In addition, we observed sleep-related and circadian activity rhythm effects generated by the dynamic ambient bedroom lighting system. Those effects were likely not caused by differences in pharmacological and non-pharmacological inpatient treatments in the two intervention groups.

Under dynamic lighting, the timing of sleep phases was altered. Patients generally woke up earlier by 20 min and, with a delay of one week, also started sleep 33 min earlier. Moreover, and independent of treatment time, depressed patients showed shorter waking periods during nighttime sleep by 15 min under dynamic lighting and slept 11 min longer in treatment week 1 and 27 min longer in treatment week 2.

Sleep disturbances are highly prevalent in patients with depressive disorders. Research has also shown a bidirectional association between sleep disturbances and depression^46^. Thus, improving sleep at the beginning of treatment is of high importance^47^.

In a recently published review which summarized effects of antidepressants in depressed patients, Tazawa et al.^48^ could show that a successful pharmacotherapy is accompanied with sleep-related effects such as shortened sleep onset latency and increased sleep efficiency (i.e., reduced nighttime activity levels and waking periods). In the present study dynamic lighting generated acute sleep-related effects within the first two inpatient treatment weeks.

Research further showed that depressive disorders are associated with circadian disturbances such as a phase delay in circadian rhythms^49^. In addition, a delayed peak in daytime activity is typically found in periods of acute depression^50^. One suspected mechanism of action of morning light therapy is its potential to phase advance the circadian system^2^. This effect has already been shown in people with seasonal affective disorder^51^. However, to date there is inconclusive evidence that phase shifting of circadian rhythms is the mechanism of action in the treatment of non-seasonal depression^1^.

In the present study, with some delay (in the second treatment week), the onset times of the daily most active and less active periods occurred sooner under dynamic bedroom lighting indicating that the intervention not only has influenced nighttime sleep but also circadian activity rhythm parameters. It should be mentioned, however, that sleep problems and circadian disturbances are mutually dependent^52^ and thus reported light effects on the circadian activity rhythm are not independent from light effects on nighttime sleep parameters.

Our study found no effects on inter-daily stability and intra-daily variability of activity rhythms, which is in line with results from a systematic review of Burton et al.^53^. It can be assumed that hospitalization and consequently the social rhythm imposed on patients’ daily activities may have masked potential light effects on the stability and variability of daily physical activity cycles.

Altered physical activity is a core feature of depression^47^ and depressed patients often show lower daytime activity levels^50,53^ and a damped circadian activity profile^47^. During the course of treatment, these physical activity parameters usually improve^53^. We found similar results in our study. Irrespective of bedroom lighting, patients’ daytime physical activity level and their daily activity rhythm amplitude improved over time, indicating a general inpatient treatment response.

In this study, patients under dynamic and standard bedroom lighting were prescribed equivalent daily doses of antidepressant, antipsychotic and sedative medication.

It is well documented that only about half of all depressed patients respond to antidepressant medication and about one third experience remission of symptoms^54^. Moreover, clinical response to antidepressant medication can often be observed after weeks^55^, which is problematic for adherence, particularly because significant side effects of antidepressant medication (e.g., digestive problems, appetite disturbances, sleep problems, dizziness, or agitation) frequently occur at the initiation of pharmacotherapy. Consequently, supplementary interventions with a fast antidepressant response are an important objective.

For a more immediate relief of specific symptoms in depressed patients (e.g., distress, sleeplessness and restlessness), sedatives and antipsychotics are frequently prescribed^56,57^. It was recently shown that combination therapy (benzodiazepines + antidepressants) reduce depression severity in the early phase of treatment (1–4 weeks) compared to antidepressants alone^58^. In addition, benzodiazepines ameliorate symptoms of insomnia efficiently. However, administration must be balanced judiciously against possible harms^59^.

There are also well recognized non-pharmacological interventions with immediate response in antidepressant treatment, such as electroconvulsive therapy^60^ and partial or total sleep deprivation^61^. However, these treatments are complex and can also have significant side effects. Due to its fast-acting response profile, light therapy has been proposed as further, well-tolerated treatment option for depressed patients^6^. The most recently published meta-analysis confirms the effectiveness of light therapy in non-seasonal depression but also states that more research is needed in severely depressed patients^63^. In addition, there is first evidence that a combination of antidepressants and bright light therapy appear particularly efficacious^62^. The present study provides initial evidence of a fast response in physical activity parameters of depressed inpatients to ambient light treatment.

We did not observe an intervention effect on the length of hospitalization, although other studies have shown increased sunlight exposure during the day to reduce inpatient treatment duration in severely depressed patients^64–67^.

This study has several limitations. First, sample size was small. Although we included 58 patients in our study, only data from 30 subjects could be analyzed. The main cause of the high drop-out rate was the poor acceptance of the wrist-worn actimeter was not well accepted. Several patients reported that the actimeter disrupted their nighttime sleep. Second, no further objective and subjective sleep measure (e.g., polysomnography, questionnaires) or circadian phase marker (e.g., melatonin or core body temperature) was recorded to confirm wrist actimetry results. Third, self-rated depression symptoms were obtained only at admission. Fourth, effects of DD-N lighting were observed for the first 14 treatment days only. Long-term effects of DD-N lighting during as well as after the hospital stay remain unknown. Fifth, we do not know which of the three lighting components (dawn simulation, dusk simulation, blue-depleted nighttime lighting) had contributed to which extent to the reported sleep-related and circadian light effects. Sixth, the lighting control system was not able to store usage times of the luminaires. Future studies should pay particular attention to recording the usage times of the lights in bedrooms and to include this data in the data analysis.

To conclude, our pilot study succeeded in demonstrating beneficial effects of dynamic bedroom lighting for inpatients with moderate to severe depression, with rapid effects on sleep and circadian activity rhythm parameters. Larger studies are warranted to establish dynamic ambient lighting as an effective treatment option.

## Supplementary Information

Supplementary Information.

## Data Availability

Aggregated data is available from the corresponding author upon request.
