# Supplementary material for: Effects of dynamic bedroom lighting on measures of sleep and circadian rest-activity rhythm in inpatients with major depressive disorder
Source: Sci Rep. 2022 Apr 12;12:6137. doi: 10.1038/s41598-022-10161-8 (PMC9005730; doi:10.1038/s41598-022-10161-8)
Supplement: Supplementary file 1 — Supplementary Information. [file 41598_2022_10161_MOESM1_ESM.docx]

**Manuscript title:**

**Effects of dynamic bedroom lighting on measures of sleep and circadian rest-activity rhythm in inpatients with major depressive disorder**

Markus Canazei*^1^, Johannes Weninger^2^, Wilfried Pohl^2^, Josef Marksteiner^3^, Elisabeth M Weiss^1^

**Institutional affiliation:**

^1^ Department of Psychology, University of Innsbruck, Innrain 52f, 6020 Innsbruck, Austria

^2^ Research department, Bartenbach GmbH, Rinnerstrasse 14, 6071 Aldrans, Austria

^3^ Abteilung Psychiatrie und Psychotherapie A, Regional Psychiatric Hospital, 6060 Hall in Tirol, Austria

**Supplementary Materials**

Figure S1: **Room lighting during the day (DD-N-lighting system).**


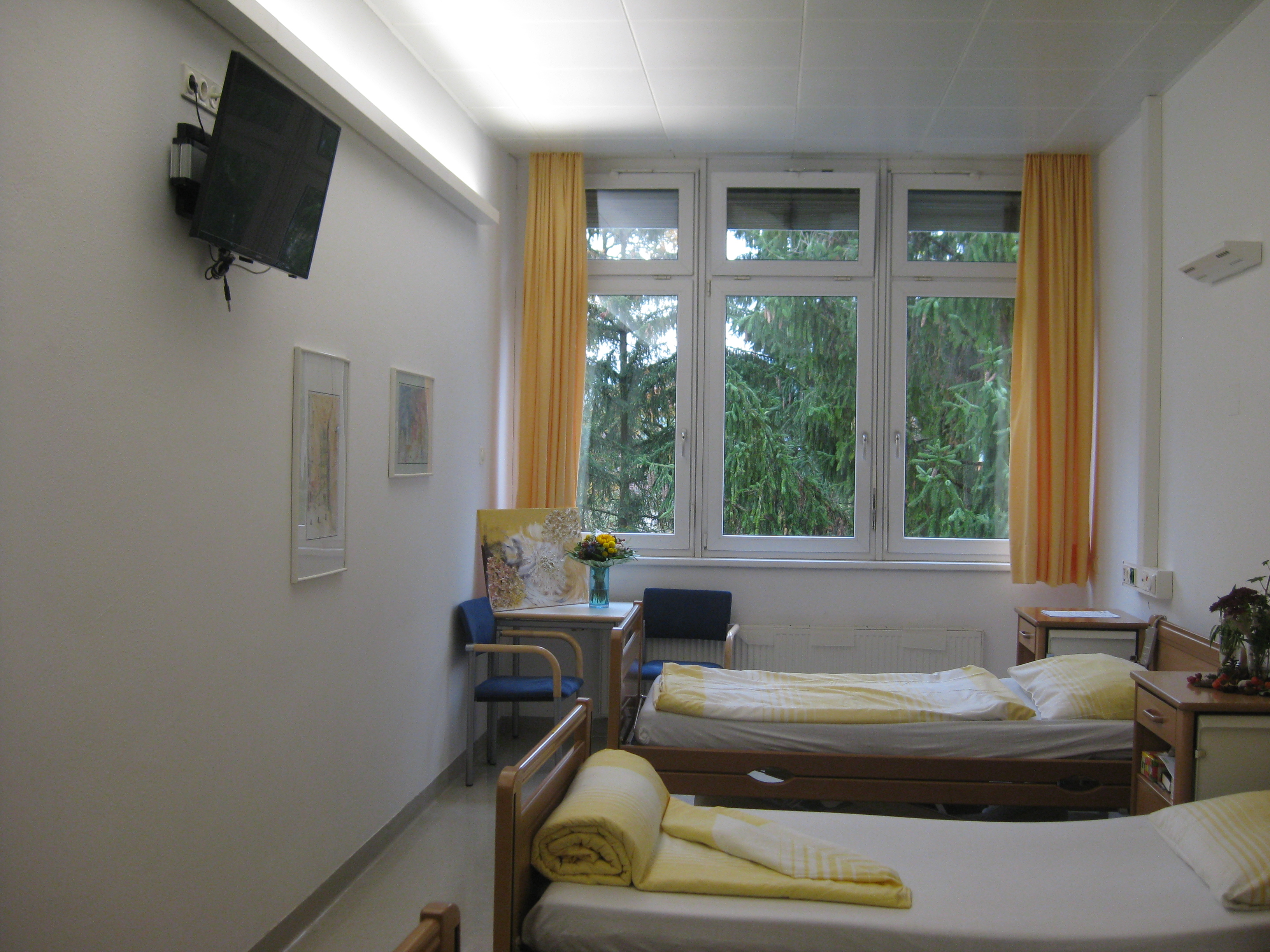


Note: ambient lighting with neutral-white light (3588 Kelvin) is switched on (the white luminaire is placed on the left upper wall); each bed was additionally equipped with a reading light (shown on the right upper wall; switched off in the photo); for medical examinations, the staff used a special function of the reading light.

Figure S2: **Room lighting with the reading lights (DD-N lighting system).**


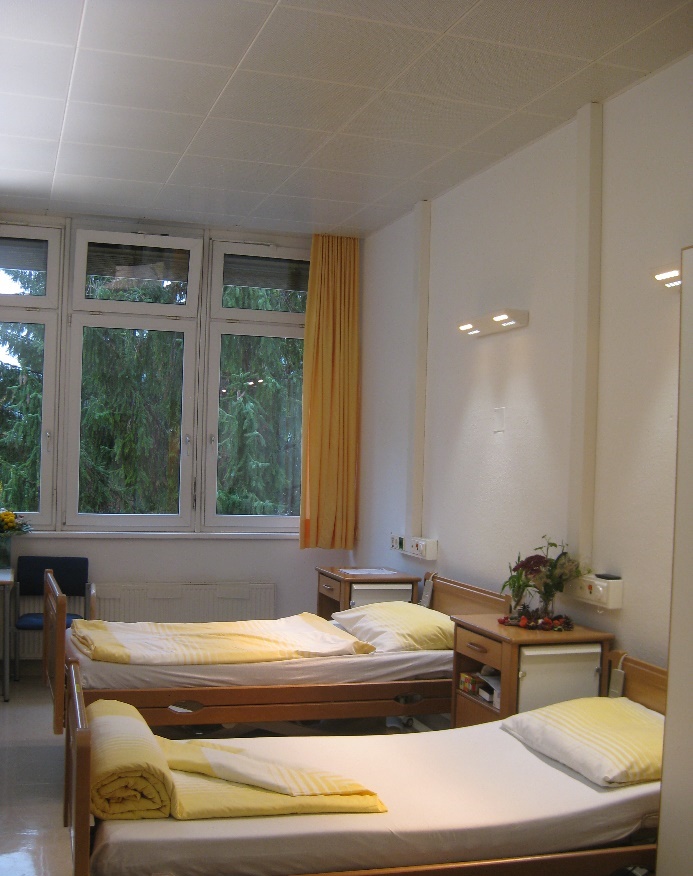


Note: the reading lights are shown on the right upper wall. The white-colored light switches for the reading lights are installed on the ride side of the beds.

Table S3: **Tabulated light spectra.**

Night* Evening (22:00h)* Day* Standard lighting

380 6,676E-07 3,338E-06 6,181E-06 4,846E-05

385 6,676E-07 3,338E-06 6,181E-06 4,846E-05

390 6,676E-07 3,338E-06 6,181E-06 4,846E-05

395 6,676E-07 3,338E-06 2,557E-05 4,846E-05

400 7,080E-07 3,540E-06 8,083E-06 1,220E-04

405 2,713E-07 1,357E-06 2,397E-05 1,390E-04

410 1,125E-07 5,626E-07 4,626E-05 1,390E-04

415 3,165E-06 1,583E-05 1,132E-04 2,127E-04

420 6,323E-06 3,162E-05 1,972E-04 3,403E-04

425 8,521E-06 4,261E-05 3,718E-04 3,575E-04

430 1,719E-05 8,594E-05 6,293E-04 8,221E-04

435 2,271E-05 1,136E-04 8,837E-04 3,167E-03

440 2,792E-05 1,396E-04 1,187E-03 7,774E-04

445 4,256E-05 2,128E-04 1,789E-03 7,427E-04

450 6,108E-05 3,054E-04 2,450E-03 8,098E-04

455 6,645E-05 3,323E-04 2,368E-03 8,060E-04

460 5,923E-05 2,961E-04 1,803E-03 7,866E-04

465 5,353E-05 2,676E-04 1,406E-03 7,384E-04

470 5,264E-05 2,632E-04 1,205E-03 6,974E-04

475 5,069E-05 2,534E-04 1,016E-03 6,632E-04

480 5,093E-05 2,546E-04 1,008E-03 9,558E-04

485 5,866E-05 2,933E-04 1,085E-03 2,091E-03

490 6,497E-05 3,248E-04 1,239E-03 1,680E-03

495 7,681E-05 3,841E-04 1,426E-03 1,084E-03

500 8,899E-05 4,450E-04 1,618E-03 6,606E-04

505 1,021E-04 5,105E-04 1,815E-03 4,591E-04

510 1,117E-04 5,586E-04 1,984E-03 4,306E-04

515 1,208E-04 6,040E-04 2,124E-03 3,703E-04

520 1,281E-04 6,407E-04 2,217E-03 3,476E-04

525 1,408E-04 7,042E-04 2,358E-03 3,441E-04

530 1,493E-04 7,464E-04 2,490E-03 4,942E-04

535 1,660E-04 8,302E-04 2,595E-03 1,421E-03

540 1,791E-04 8,956E-04 2,698E-03 7,682E-03

545 1,937E-04 9,685E-04 2,827E-03 9,118E-03

550 2,154E-04 1,077E-03 2,968E-03 2,730E-03

555 2,313E-04 1,157E-03 3,073E-03 8,511E-04

560 2,516E-04 1,258E-03 3,127E-03 5,309E-04

565 2,805E-04 1,403E-03 3,235E-03 4,727E-04

570 3,056E-04 1,528E-03 3,340E-03 4,080E-04

575 3,351E-04 1,675E-03 3,424E-03 1,154E-03

580 3,679E-04 1,839E-03 3,478E-03 1,977E-03

585 3,950E-04 1,975E-03 3,544E-03 2,399E-03

590 4,248E-04 2,124E-03 3,625E-03 1,909E-03

595 4,517E-04 2,258E-03 3,612E-03 1,405E-03

600 4,720E-04 2,360E-03 3,610E-03 1,149E-03

605 4,879E-04 2,440E-03 3,556E-03 1,674E-03

610 4,961E-04 2,480E-03 3,506E-03 1,141E-02

615 5,067E-04 2,534E-03 3,438E-03 3,608E-03

620 5,029E-04 2,514E-03 3,341E-03 2,064E-03

625 4,925E-04 2,463E-03 3,185E-03 2,278E-03

630 4,779E-04 2,390E-03 3,020E-03 2,091E-03

635 4,613E-04 2,307E-03 2,841E-03 7,115E-04

640 4,420E-04 2,210E-03 2,636E-03 6,143E-04

645 4,187E-04 2,094E-03 2,458E-03 6,979E-04

650 3,952E-04 1,976E-03 2,273E-03 1,007E-03

655 3,643E-04 1,821E-03 2,073E-03 7,260E-04

660 3,366E-04 1,683E-03 1,866E-03 7,636E-04

665 3,120E-04 1,560E-03 1,685E-03 6,438E-04

670 2,805E-04 1,403E-03 1,493E-03 5,771E-04

675 2,549E-04 1,275E-03 1,343E-03 5,179E-04

680 2,339E-04 1,170E-03 1,205E-03 5,696E-04

685 2,118E-04 1,059E-03 1,063E-03 6,855E-04

690 1,880E-04 9,400E-04 9,426E-04 5,580E-04

695 1,692E-04 8,459E-04 8,484E-04 4,398E-04

700 1,506E-04 7,531E-04 7,288E-04 4,427E-04

705 1,330E-04 6,649E-04 6,448E-04 1,460E-03

710 1,174E-04 5,870E-04 5,560E-04 1,393E-03

715 1,025E-04 5,127E-04 4,907E-04 4,342E-04

720 9,083E-05 4,542E-04 4,185E-04 3,210E-04

725 7,987E-05 3,993E-04 3,701E-04 3,293E-04

730 6,940E-05 3,470E-04 3,209E-04 3,261E-04

735 6,192E-05 3,096E-04 2,748E-04 3,622E-04

740 5,220E-05 2,610E-04 2,436E-04 3,789E-04

745 4,603E-05 2,301E-04 2,079E-04 3,672E-04

750 3,970E-05 1,985E-04 1,878E-04 3,442E-04

755 3,414E-05 1,707E-04 1,566E-04 6,760E-04

760 2,973E-05 1,487E-04 1,297E-04 8,998E-04

765 2,621E-05 1,310E-04 1,149E-04 3,433E-04

770 2,407E-05 1,203E-04 1,091E-04 4,177E-04

775 1,953E-05 9,766E-05 9,476E-05 3,373E-04

780 1,687E-05 8,435E-05 8,083E-05 7,916E-04

Note: * = light spectra with DD-N lighting system

Figure S4: **Sleep Analyses.**


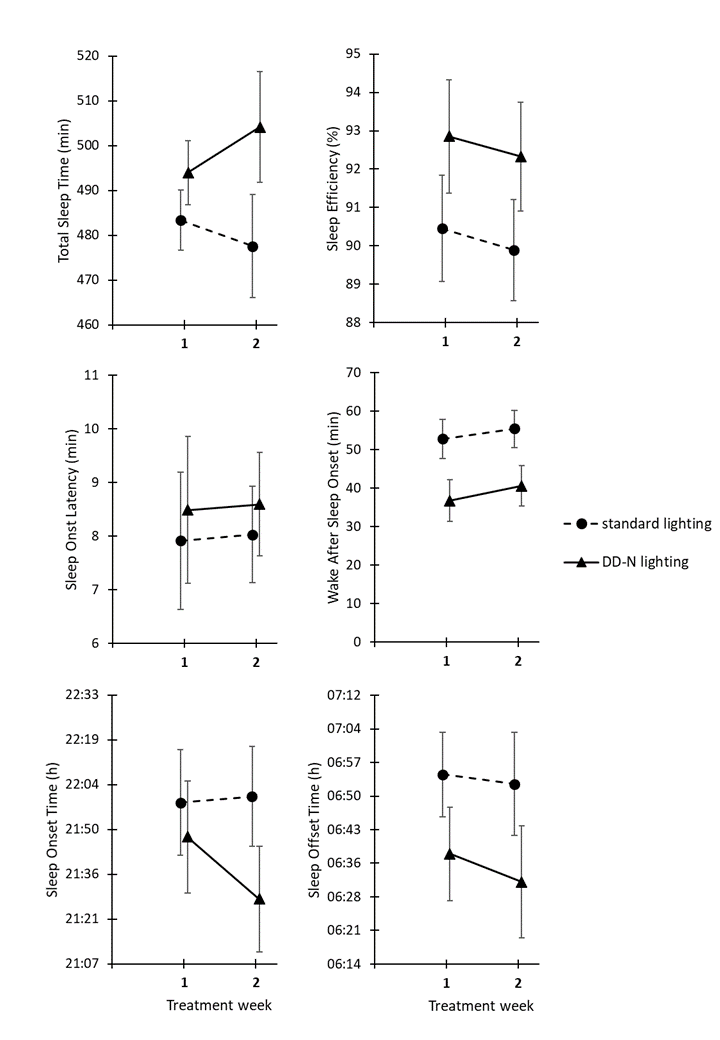


Table S5: **Comparison of sleep parameters (first 3 treatment days) between the two intervention groups**.

|  | Patients in bedrooms with standard lighting  (n=16) | Patients in bedrooms with DD-N lighting (n=14) | Statistics* |
| --- | --- | --- | --- |
| Total sleep time [min] | 485 ± 70 | 490 ± 43 | *p* = .819 |
| Sleep efficiency [%] | 88 ± 5 | 91 ± 3 | *p* = .107 |
| Sleep onset latency [min] | 9 ± 3 | 7 ± 2 | *p* = .090 |
| Wake after sleep onset [min] | 55 ± 25 | 40 ± 19 | *p* = .114 |
| Sleep onset time (hh:mm) | 21:43 ± 61 min | 21:56 ± 38 min | *p* = .466 |
| Sleep offset time (hh:mm) | 06:40 ± 26 min | 06:46 ± 48 min | *p* = .707 |

Note: *two-way mixed analysis of variances (ANOVAs) with the between-subjects factor light intervention and the within-subjects factor treatment week were used.

Figure S6a: **Non-Parametric Circadian Rest-Activity Rhythm Analyses (Part 1).**


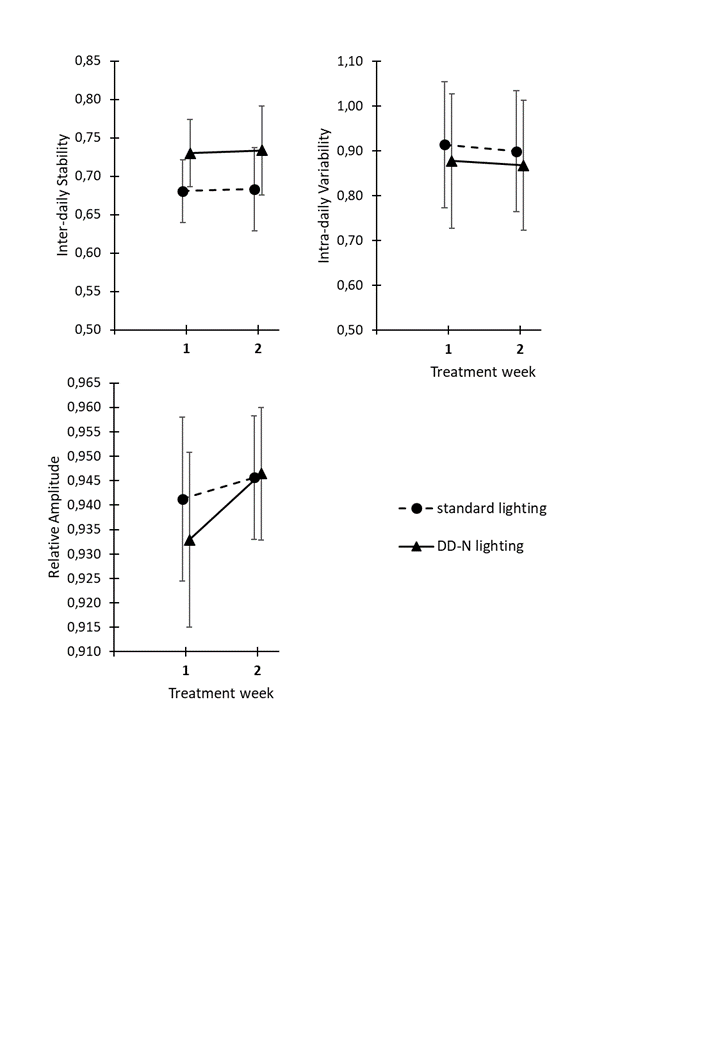


Figure S6b: **Non-Parametric Circadian Rest-Activity Rhythm Analyses (Part 2).**

**
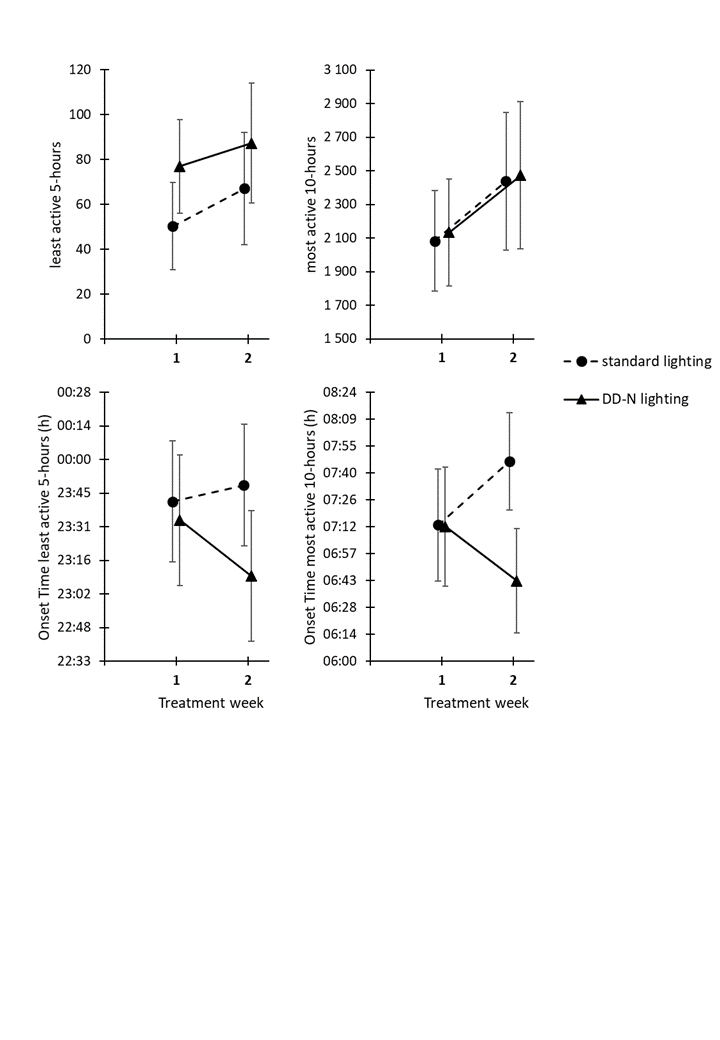
**
